# Clinical, biochemical features and functional outcome of patients with SARS-CoV-2-related subacute thyroiditis: a review

**DOI:** 10.1007/s12020-022-03247-w

**Published:** 2022-11-17

**Authors:** N. Viola, A. Brancatella, D. Sgrò, F. Santini, F. Latrofa

**Affiliations:** Endocrinology Unit I, Department of Clinical and Experimental Medicine, University Hospital of Pisa, Pisa, Italy

**Keywords:** Subacute thyroiditis, SARS CoV-2, COVID-19

## Abstract

**Introduction:**

SARS CoV-2 infection involves many organs and systems, including the thyroid, in which it manifests itself as subacute thyroiditis (SAT). After our first description of SAT due to SARS-CoV2 infection, other reports have confirmed the correlation between SARS-CoV-2 and SAT. We review the cases of SAT associated with COVID-19 to highlight its peculiar clinical and biochemical features, including its outcome and what it has added to our understanding of SAT.

**Results:**

We have reviewed 24 articles, for a total of 69 cases of SAT related to SARS-CoV2 infection. All had neck pain, whereas thyrotoxicosis was documented in 68/68 who had their thyroid function checked. Ultrasound, performed in 67 patients, was typical of SAT in 65 and low uptake at scintigraphy was demonstrated in all 12 evaluated patients. Patients had a prompt response to the anti-inflammatory and/or glucocorticoid therapy, as expected in SAT. The rate of hypothyroidism was higher (36.5%) in COVID-19-related SAT compared to that observed in the pre-COVID era (10%).

**Conclusions:**

Clinical, biochemical, and instrumental features of SAT related to SARS-CoV2 are like those observed in SAT cases reported prior to COVID-19 pandemic, but it appears more severe.

## Introduction

Subacute thyroiditis (SAT) was first described in 1895 by Mygind as an inflammation of a previously normal thyroid gland without abscess formation [1]. SAT is caused by a viral infection or a post-viral inflammatory process occurring in genetically predisposed subjects [2]. Several viruses have been associated with SAT, namely mumps virus, coxsackievirus, enterovirus, echovirus, adenovirus, influenza virus, Epstein-Barr virus, hepatitis E virus, cytomegalovirus, dengue virus, rubella virus and, very recently, SARS-CoV2 [3–7]. As of June 23, 2022, the World Health Organization has reported more than 538 million cases of COVID-19, with a death toll of more than 6.3 million. Following the first case of SAT related to SARS-CoV-2 infection reported by our group [7], other case reports, case series and studies were published [8–30]. Data from the observational study, investigating patients admitted to intensive care units because of COVID-19 and describing atypical, “painless”, presentation were excluded [31]. Another study identified two subsets of SAT: a painless type, more common after recovery from COVID-19, characterized by symptoms of thyrotoxicosis, and a painful type, more frequent long after recovery from COVID-19, presenting with a less severe thyrotoxicosis [30]. Only patients with pain reported in this study were included in this review. Some papers have already reviewed the first studies that described SAT cases related to COVID-19 infection. However, we have recently published a paper investigating a large cohort of COVID-19-related SAT, and, for the first time, we compared the features of this group with those observed in SAT patients prior to COVID-19 pandemic [29]. We, therefore, decided to write a more complete review, including our and other recent papers, to better characterize SAT due to SARS-CoV2.

## Methods

### Search strategy

Online databases including PubMed/Medline and Google Scholar were searched for articles published until May 30, 2022. Search strategy was based on the following keywords: COVID-19 or SARS-CoV-2 and Subacute Thyroiditis or De Quervain’s Thyroiditis. To identify additional studies, references of the retrieved articles were also screened. The authors declare that the study selection was conducted in the absence of any commercial or financial relationships that could be taken as a potential conflict of interest.

### Study selection

The relevant published articles on this topic were mainly case reports and case series. We excluded cases related to COVID vaccination. Moreover, our search focused on studies reporting typical cases of SAT, i.e., characterized by neck pain and thyrotoxicosis. Therefore, patients diagnosed with “atypical” thyroiditis were excluded. In addition, we did not include in this review paper evaluating COVID-19 infection and low T3 syndrome. All those included in the final analysis were in English language. Data are reported in Table 1.

**Table 1:** Demographic features, symptoms, laboratory findings, ultrasound characteristics, and outcome of SARS-CoV-2 related SAT. FT4 and FT3 are reported in pmol/L, TSH is reported in mUI/L, Tg in mcg/L, ESR in mm/h, RCP in mg/dL

Author	Sex	Age	SAT symptoms	FT4	FT3	TSH	Tg	TgAb	TPOAb	TRAb	ESR	RCP	SAT pattern at US	Outcome
[7] Brancatella	F	18	Neck pain, tachycardia	27.2	8.7	<0.005	5.6	Pos	Neg	Neg	90	6.9	Yes	Euthyroidism
[9] Asfuroglu	F	41	Neck pain, fever	25.7	7.7	<0.008	-	Neg	Neg	Neg	134	10.1	Yes	-
[10] Mattar	M	34	Neck pain, fever	41.8	13.4	<0.01	-	-	Neg	Neg	-	12.2	Yes	Euthyroidism
[11] C. Barrera	F	37	Neck pain, fatigue	20.6	-	<0.01	-	Neg	Neg	-	72	6.6	-	Euthyroidism
[12] Khatri	F	41	Neck pian, weight loss	60.3	-	<0.01	-	-	Pos	Neg	107	36.4	Yes	-
[13] San Juan	F	47	Neck pain, swelling	21.6	-	0.05	-	Neg	Neg	Neg	-	5	Not	Hypothyroidism
[14] Guven	M	49	Neck pain, swelling	56.5	6.6	<0.05	-	Neg	Neg	Neg	80	7.7	Yes	-
[15] Chong	M	37	Neck pain, tachycardia	23	-	0.01	-	Neg	Neg	Neg	-	-	Not	Hypothyroidism
[16] Ruggeri	F	43	Neck pain, palpitations	34.6	10.8	0.006	188	Neg	Neg	Neg	60	8.8	Yes	Euthyroidism
[17] Chakraborty	M	58	Neck pain, tachycardia	24.7	8.7	<0.005	-	-	-	-	110	16.6	Yes	Hypothyroidism
[18] Sohrabpour														
Case1	F	26	Neck pain, palpitations	19.5	18.9	0.07	-	-	-	-	70	28	Yes	Euthyroidism
Case 2	F	37	Neck pain, palpitations	22.3	25.4	<0.01	-	-	-	-	38	56	Yes	Euthyroidism
Case 3	M	35	Neck pain, palpitations	24.7	19.3	0.12	-	-	-	-	45	18	Yes	Euthyroidism
Case 4	F	41	Neck pain, palpitations	21.9	23.7	<0.01	-	-	-	-	83	43	Yes	Euthyroidism
Case 5	M	52	Neck pain, palpitations	26.7	21.6	0.17	-	-	-	-	76	51	Yes	Euthyroidism
Case 6	F	34	Neck pain, palpitations	18.4	18.1	0.23	-	-	-	-	39	23	Yes	Euthyroidism
[8] Brancatella														
Case 1	F	38	Neck pain, fever, palpitations	29.3	8	0.1	75.3	Neg	Neg	Neg	74	11.2	Yes	Euthyroidism
Case 2	F	29	Neck pain, fever, palpitations	31	8.9	<0.001	80	Pos	Neg	Neg	110	7.9	Yes	Hypothyroidism
Case 3	F	29	Neck pain, palpitations	-	-	-	-	-	-	-	-	-	Yes	Hypothyroidism
Case 4	F	46	Neck pain, palpitations	27.8	6.9	<0.001	-	-	-	Neg	-	8	Yes	Euthyroidism
[19] de la Higuera	F	36	Neck pain, palpitations	27.9	-	0.008	-	Neg	Neg	Neg	31	1.05	-	Euthyroidism
[20] Resuli														
Case 1	F	32	Neck pain, palpitations	26.8	8.4	<0.001	-	Neg	Neg	-	65	-	Yes	Euthyroidism
Case 2	F	25	Neck pain, palpitations	28.1	9.6	<0.001	-	Neg	Neg	-	58	-	Yes	Euthyroidism
Case 3	F	45	Neck pain, palpitations	43.1	14.2	<0.001	-	Neg	Neg	-	70	-	Yes	Euthyroidism
Case 4	F	29	Neck pain, palpitations	38.1	11.3	<0.001	-	Neg	Neg	-	65	-	Yes	Euthyroidism
Case 5	F	21	Neck pain, weight loss	43.5	16.2	<0.001	-	Neg	Neg	-	80	-	Yes	Euthyroidism
[21] Semikov														
Case 1	F	46	Neck pain, tachycardia, fever	43.8	-	<0.008	-	-	Neg	-	47	3.32	Yes	Euthyroidism
Case 2	F	46	Neck pain, tachycardia	41	-	0.005	-	-	Neg	-	32	-	Yes	Euthyroidism
[22] Álvarez	F	46	Neck pain, fever	28	-	0.11	-	-	Pos	-	65	-	Yes	Hypothyroidism
[23] Sato	F	31	Neck pain, fever	41	11	<0.001	-	Pos	-	-	93	3.6	Yes	Euthyroidism
[24] Stasiak														
Case 1	M	50	Neck pain, fever, tachycardia	-	-	-	-	Neg	Neg	Neg	52	9	Yes	Euthyroidism
Case 2	F	39	Neck pain, fever, Tachycardia	99	33	< 0.005	-	Neg	Neg	Neg	59	60	Yes	Euthyroidism
Case 3	F	55	Neck pain	30.8	7.9	0.01	-	Pos	Neg	Neg	140	4.98	Yes	-
Case 4	F	57	Neck pain, fever Tachycardia	30.1	8.04	0.07	-	Neg	Neg	Neg	117	4.71	Yes	-
[25] Salehi	M	55	Neck pain, fever, palpitations	15,8	19	0.29	-	-	-	-	121	9.26	Yes	Euthyroidism
[26] Elawadi	F	33	Neck pain	-	-	0.04	-	Neg	Neg	-	-	-	Yes	-
[27] Ullah	M	30	Neck pain,palpitations, tachicardia	24	5	0.005	-				88	12	Yes	Euthyroidism
[28] Bahçecioğlu (*n* = 12)	F (*n* = 5) M (*n* = 7)	49	Neck pain (*n* = 12) Fever (*n* = 3)	27.9	8.1	0.015	-	-	-	-	47.4	49.2	Yes (*n* = 12)	-
[29] Brancatella (*n* = 14)	F (*n* = 14)	34	Neck pain (*n* = 14) Fever (n = 13)	26.9	7	0.02	155	-	-	-	91	8.5	Yes (*n* = 14)	Hypothyroidism (*n* = 11) Euthyroidism (*n* = 3)
[30] Mondal (*n* = 6)	F (*n* = 4) M(*n* = 2)	51	Neck pain (*n* = 6), fever (*n* = 3) palpitations (*n* = 2)	21.2	5.1	0.02	-	-	-	-	72.8	6.9	Yes (*n* = 6)	Hypothyroidism (n = 2) Euthyroidism (*n* = 4)

Ref. [27]: *n* = 14 plus 4 already report in ref. [19] refs. [29–31]: data are reported as mean

## Results

Our search identified 24 articles—16 case reports, 5 case series, 2 cross-sectional study, 1 prospective study—, for a total of 69 patients. The mean age of patients was 39.8 years (range 18–69 years) (Table 1). Of 69 patients, 49 were females (71%), and 20 males (29%). Twenty cases were reported in Italy, 19 in Turkey, 8 in Iran, 4 in Poland, 2 in USA, Russia and Spain, 1 in Singapore, Mexico, the Philippines, Pakistan, Egypt, and Japan. Family history was reported in 10 patients. One patient had a family history of thyroid disease, whereas 2 had a goiter long before the onset of symptoms related to SAT. The interval between the start of COVID-19 illness and the appearance of SAT symptoms was 32 days (mean) (range: 0–224 days). Among 36 patients in whom data about the diagnosis of COVID-19 were available, it was obtained by RT-PCR swabs in 23 patients (59%) and by positive serum SARS-CoV-2 IgG or IgM in 13 (41%). Neck pain was the most common symptom (69/69 patients). Other complaints included fatigue, fever, odynophagia and symptoms of thyrotoxicosis, i.e., palpitations, tachycardia, and weight loss. Concerning laboratory tests, thyrotoxicosis was demonstrated in 68 patients tested at the time of SAT (Table 1). Thyroglobulin (Tg), measured in 19 patients who had undetectable Tg autoantibodies (TgAbs), was always elevated. Positive TgAbs and positive thyroperoxidase autoantibodies (TPOAbs) were tested in few patients and found positive at low levels. In 28 patients in whom autoantibodies to the TSH-receptor (TRAb) were assayed, no detectable TRAb were found. Sixty-three patients had the Erythrocyte Sedimentation Rate (ESR) measured and values were high in all. C-reactive protein (CRP) values were also high in 60 patients in whom it was measured. Hypoecoic, dishomogeneous areas suggestive of SAT were found in 65 out of 67 patients who had thyroid ultrasounds. A reduced or absent uptake of the thyrotropic tracer (^131^I or ^99^Tc) was observed in 12 patients who received thyroid scintigraphy. Forty-eight patients were treated with steroids, 6 with aspirin, 4 with not specified non-steroidal anti-inflammatory drugs (NSAIDs), 4 with NSAIDs and steroids, 3 with ibuprofen, 1 with hydroxychloroquine, 1 with mefanic acid and 2 patients were left untreated. Five patients were given beta-blockers for symptoms of thyrotoxicosis. A prompt recovery from painful symptoms within few days of treatment was reported in all patients. Data on the duration of the follow-up were available in 52 patients; the median time was 8 weeks. Thyroid functional outcome at the end of follow-up was reported in 52 patients: 31 (64%) were euthyroid, 19 (36.5%) hypothyroid.

## Discussion

In 2020 SARS-CoV-2, which originated in Wuhan, China, spread quickly worldwide, emerging as the cause of a respiratory disease (COVID-19) of variable severity [32, 33]. Other tissues may be also involved in SARS-CoV-2 infection [34, 35]. SARSCoV-2 at its cellular entry recognizes receptor angiotensin-converting enzyme 2 (ACE-2), which has been demonstrated in follicular thyroid cells as well [36]. After our first report as of May 2020, several cases of SAT associated with SARS-CoV-2 infection have been described [7–30]. Furthermore, SARS-CoV2 infection has been also related to autoimmune thyroid diseases, namely Hashimoto’s thyroiditis, Graves’ disease, and Graves’ orbitopathy [37, 38]. According to the two studies evaluating this issue, the overall incidence of SAT during the COVID-19 pandemic was similar to that previously reported [29, 39]. Other studies reported an atypical form of destructive thyroiditis, with thyrotoxicosis, in 15–20% of patients [31, 40]. However, studies investigating thyroid function in patients with COVID-19 infection differ in characteristics (age, M/F ratio, ethnicity) of populations under investigation, study design, severity of COVID-19, time of evaluation of thyroid function; in addition, results may be influenced by factors interfering with both thyroid function (i.e., steroids and iodinated contrast media) and thyroid tests (heparin). Indeed, when patients with no interfering factors were analyzed separately, low TSH levels were associated with low FT3 levels in half cases and normal levels of FT4 and Tg were observed in all patients [41]. The inverse correlation between the transiently low TSH levels and CRP, cortisol, and IL-6 values was ascribed to the cytokine storm induced by SARS-Cov-2, which impacted on TSH secretion and deiodinase activity. SAT typically presents as pain localized to the anterior neck that may radiate to the jaw or the ear associated with low-grade fever, fatigue, and mild symptoms of thyrotoxicosis. Laboratory findings confirm thyrotoxicosis (suppressed TSH, high FT4) and inflammatory markers, i.e., ESR and CRP, are typically elevated. Uptake at thyroid scintigraphy is low or absent and at thyroid ultrasound poorly defined hypoechoic areas are recognized [42].

As previously reported, most cases we herein review resembled the above-mentioned features typical of SAT [43]. Indeed, as commonly seen in previous SAT series, >70% of patients were females. All patients presented with anterior neck pain, some referred also generalized fatigue, fever, and swelling. Neck pain is the cardinal, presenting feature of SAT [2]. In the largest (patients = 852) SAT cohort reported to date, all patients referred neck pain at the onset of the disease, unilateral in 68.2% and bilateral in 31.8% of cases [42]. Our observations on symptoms of COVID-19-related SAT are, as expected, similar to those reported by a recent review [43].

Cases of painless SAT have been reported in the pre-COVID period, with a prevalence of 6% [44]. A high frequency of painless SAT due to COVID-19 has also been reported by some studies [30, 31]. This high frequency was ascribed to the COVID-19-associated lymphopaenia, which would prevent lymphocytic infiltration and giant cell formation within thyroid, leading to the lack of tension of the thyroid capsule and thus absence of pain [31]. However, there are some factors that can lead to an overestimation of the proportion of patients with the painless variant of SAT. First, the concomitant anti-inflammatory and steroid therapy administered to patients suffering from Covid-19 could prevent the onset of pain. Furthermore, many patients with painless SAT were in intensive care unit and/or were suffering from severe forms; it is conceivable that many of them were unable to report pain because of their general condition and treatment. The recent finding of a copious mononuclear infiltrate forming granuloma-like structures with giant multinucleated cells, resembling those observed in previous cases of SAT, in the thyroid tissue obtained from a patient deceased from COVID-19 is against the hypothesis of a reduced lymphocytic and plasmocytic infiltration of the thyroid, resulting from lymphopenia [45]. Among patients described in this paper, most were treated with glucocorticoids, with an excellent symptomatic response, a result similar to that reported in SAT described in the pre-COVID period. Regarding the functional outcome of the thyroid, 19 of the 52 patients (36.5%) for whom data on thyroid function were available 4 weeks or more after the diagnosis of SAT, became hypothyroid. This figure is significantly higher compared to data collected in the pre-Covid-19 series of SAT, which reported an incidence rate of hypothyroidism of 10% [46].

A recent review reported that SAT clinical presentation in COVID-19 patients is similar to SAT cases reported prior to COVID-19 pandemic [43]. However, the authors admitted that the size and quality of paper they reviewed were poor, with only case series and case report published at that time [43]. We have recently published the results of a cross-sectional study including the cases of SAT observed in the year 2020 and those observed prior to the SARS-CoV-2 pandemic (years 2016-2019). Most SAT cases observed in the period 2016–2019 occurred in the third quarter of the year, similarly to previous reports, whereas in 2020 most cases were recorded in the second and fourth quarters, within a month from the two main SARS-COV-2 outbreaks in Italy. In that study we observed that SAT occurring in the second and fourth quarter of 2020 were characterized by higher levels of FT4, CRP, Tg, and ESR and resulted in hypothyroidism in >80%. All these findings highlight that SAT cases induced by SARS-CoV-2 are more severe than those observed in the pre-COVID19 period, caused by other viruses, thus suggesting that the causative virus influences the severity of SAT and the risk of developing hypothyroidism.

In conclusion, SAT induced by COVID-19, is similar to SAT caused by other viral agents regarding clinical manifestations, i.e., neck pain, laboratory and ultrasound features as well as in terms of response to medical treatment. At variance SAT caused by SARS-CoV-2 is characterized by a more severe inflammatory process compared to SAT due to other viral agents commonly associated with it; this can lead to a more severe clinical picture and higher rates of hypothyroidism.
